# Determination of heme in microorganisms using HPLC-MS/MS and cobalt(III) protoporphyrin IX inhibition of heme acquisition in *Escherichia coli*

**DOI:** 10.1007/s00216-017-0610-5

**Published:** 2017-10-17

**Authors:** Jonas Fyrestam, Conny Östman

**Affiliations:** Division of Analytical and Toxicological Chemistry, Department of Environmental Science and Analytical Chemistry, Stockholm University, Svante arrheniusväg 16C, 106 91 Stockholm, Sweden

**Keywords:** Heme acquisition, Heme analysis, *Escherichia coli*, HPLC-MS/MS, Antimicrobial resistance, Porphyrins

## Abstract

One of the main threats to the achievements in modern medicine is antimicrobial resistance. Molecular targeting of bacterial acquisition mechanisms of heme has been suggested to be an alternative to antibiotics. In the present study, HPLC-MS/MS combined with a simple clean-up based on liquid-liquid extraction has been developed and evaluated for simultaneous determination of heme and porphyrin heme precursors in microorganisms. Experimental design was used to optimize the extraction parameters, to obtain a method with high recovery, low matrix effects, and high precision. The effects of additives in the culture medium on the biosynthesis of heme were studied using *Escherichia coli* as a model microorganism. 5-Aminolaevulinic acid and hemin increased the heme concentration in *E. coli* by a factor of 1.5 and 4.5, respectively. Addition of 5-aminolaevulinic acid bypassed the *E. coli* negative feedback control of heme biosynthesis, which led to high amounts of intracellular porphyrins. The high heme concentration obtained when hemin was used as a culture additive shows that *E. coli* has an uptake of heme from its surroundings. In contrast, addition of cobalt protoporphyrin IX to the growth medium reduced the amount of heme in *E. coli*, demonstrating this compound’s ability to mimic real heme and inhibit the heme acquisition mechanisms.

**Electronic supplementary material:**

The online version of this article (10.1007/s00216-017-0610-5) contains supplementary material, which is available to authorized users.

## Introduction

Increasing antimicrobial resistance among pathogens has been pointed out by the World Health Organization to be a problem so serious that it threatens the achievements of modern medicine [1]. In recent years, it has been proposed that targeting the mechanisms that take part in the acquisition of iron could be an important complement to antibiotics. In the long run, this could help to decrease the amount of antibiotics used which is the main reason for increased antimicrobial resistance against antibiotics worldwide [2–9].

**Fig. 1:**
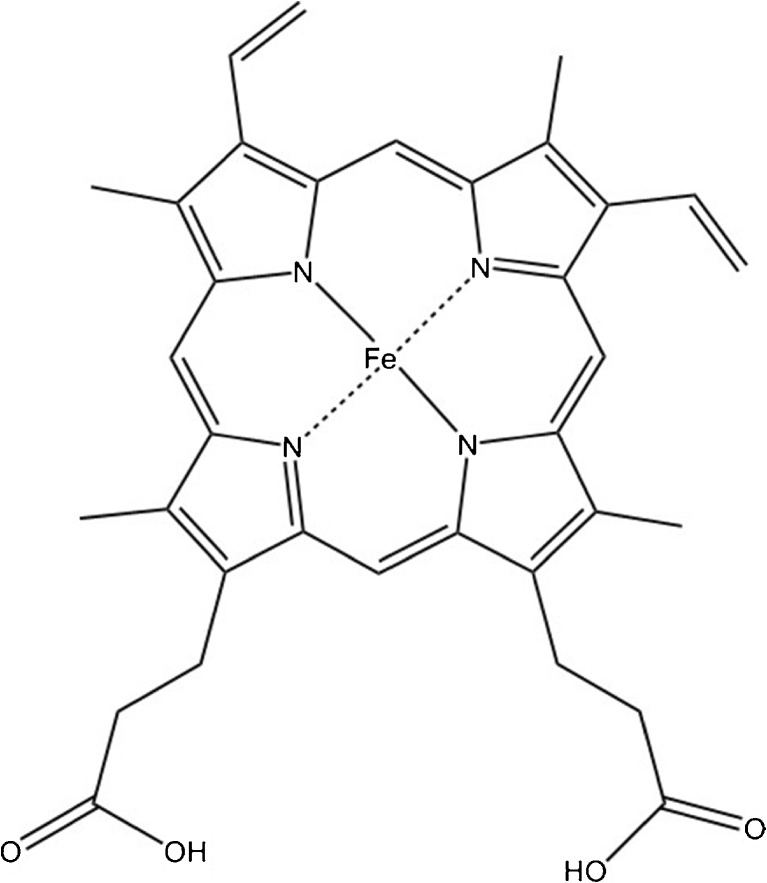
Chemical structure of heme

Iron acts as a crucial cofactor in many important biological processes such as respiration and DNA synthesis and is essential for most living organisms [10, 11]. Pathogenic bacteria are no exception, and to cause a disease, they need to acquire iron from their hosts [12]. In vertebrates, the majority of iron is present bound to a porphyrin ring, i.e., heme (Fig. 1) [2]. Vertebrates have developed strategies to limit bacterial access of free iron during an infection, a process often known as iron-withholding [13]. Transferrin and lactoferrin, two proteins with a high affinity for iron, are synthesized in excess during the first stage of an infection to reduce the levels of free iron available for pathogens that are required for their survival. These mechanisms are reducing free iron to negligible amounts, below 10^−18^ M [2]. As a response to these iron-withholding processes, bacteria have developed different strategies to acquire iron from their host. Pathogens start to produce and release compounds into the extracellular medium to scavenge heme or iron from a number of sources. Gram negative bacteria produce and excrete proteins (hemophores) that bind to heme. These proteins have high affinity for heme and they return to specific receptors located in the outer membrane of the bacteria [14]. Bacteria also have heme acquisition systems with receptors that recognize heme and transport it into the cell via ATP-binding cassette transporters [15, 16].

A number of non-iron metalloporphyrins have previously shown to be potent antimicrobial compounds [9, 17–19]. A putative cause of this toxicity to pathogens is that these compounds chemically mimic real heme. In this way, metalloporphyrins can be a substrate for the heme acquisition mechanisms and taken up by the cell. Inside the cell, these molecules are partitioned into the cell membrane, displacing heme and inhibiting respiration [17, 19].

Accurate determinations of heme are essential to widen our understanding of how different microbes acquire heme. Although heme is of great importance for microbial survival, there are currently no validated methods for selective determination of trace levels of heme in microorganisms. Often, unspecific methods are used such as UV-Vis absorption spectroscopy [20]. Heme has previously been determined by HPLC in plant cells and cyanobacteria [21], as well as in phyto- and bacterioplankton [22], but the analytical methods used in these studies lack sufficient evaluation to accurately determine heme.

The aim of this study was to develop and evaluate an analytical method for the extraction, clean-up, and analysis of heme utilizing HPLC-MS/MS using *Saccharomyces cerevisiae* and *Escherichia coli* as model microorganisms. The method was applied to determine heme in *E. coli* and its relation to microbial synthesis and/or acquisition of heme when grown using different culturing conditions.

## Experimental

### Chemicals

Formic acid (≥ 98%), tris(hydroxymethyl)aminomethane (Tris), ferrous sulfate, magnesium sulfate, and separate standards of protoporphyrin IX (purity ≥ 95%), protoporphyrin IX cobalt chloride, 5-aminolaevulinic acid hydrochloride (purity ≥ 97%), and hemin from porcine (purity ≥ 97%) were obtained from Sigma-Aldrich (Schnelldorf, Germany). HPLC-grade methanol, acetone, and acetonitrile were purchased from Rathburn Chemicals Ltd. (Walkerburn, Scotland). Analytical-grade hydrochloric acid (37%) and dimethylformamide (DMF) were obtained from VWR International (Fontenay-sous-Bois, France). Ethylenediaminetetraacetic acid disodium dihydrate salt (EDTA) and sodium chloride of reagent grade were acquired from Scherlab S.L. (Sentmenat, Spain). A Synergy 185 water purification system from Millipore (Molsheim, France) was used to produce deionized water at 18 MΩ cm. Porphyrin acid chromatographic marker kit (CMK-1A) containing 10 ± 1 nmol of each of six porphyrins (mesoporphyrin IX, coproporphyrin I, 5-carboxylporphyrin I, 6-carboxylporphyrin I, 7-carboxylporphyrin I, and uroporphyrin I) were obtained from Frontier Scientific Inc. (Logan, UT, USA). *S. cerevisiae* was used for method validation and it was obtained from Jästbolaget (Sollentuna, Sweden). *E. coli* NovaBlue was obtained from the Department of Biochemistry and Biophysics at Stockholm University.

### Culturing and harvest of *E. coli*

*E. coli* was cultivated in sterile Miller lysogeny broth (LB) at 37 °C. To determine the influence of different additives on heme and iron acquisition mechanisms in *E. coli*, the LB medium was supplemented with five additives: Fe(II)SO_4_, hemin, 5-aminolevulinic acid hydrochloride (5-ALA), and protoporphyrin IX cobalt chloride. The influence of cultivation time was also investigated. The different culture conditions are listed in Table 1.

**Table 1:** Different culture conditions for the cultivation of *E. coli*

Sample	Additive	Additive role in bacterial iron acquisition	Concentration of additive [M]	Cultivation time (total) [h]	Cultivation time (with additive) [h]
Control, 16 h	–	–	–	16	–
Control, 84 h	–	–	–	84	–
1	Fe(II)SO	Fe^2+^ is inserted in PPIX to form heme	50 × 10^−6^	16	16
2	5-ALA	Precursor of heme	5.0 × 10^−3^	84	24
3	Hemin	Exogenous source of heme	10 × 10^−6^	84	24
4	Co-PPIX	Inhibitor of heme acquisition	10 × 10^−6^	84	24
5	Co-PPIX	Inhibitor of heme acquisition	10 × 10^−6^	85	25
	Hemin	Exogenous source of heme	10 × 10^−6^		24

*PPIX* protoporphyrin IX, *5-ALA* 5-aminolaevulinic acid hydrochloride, *Co-PPIX* protoporphyrin IX cobalt chloride

*E. coli* was allowed to grow to stationary phase for 60 h; thereafter, the different additives were added for individual experiments and grown for additional 24 h (samples 2–5 in Table 1). In sample 5 (Table 1), cobalt protoporphyrin IX’s (Co-PPIX) ability to inhibit *E. coli* hemin acquisition was investigated. Culture was allowed to grow for 60 h, and then Co-PPIX was added and grown for an additional 1 h. Hemin was added and the culture was grown for 24 h. Estimation of sample sizes was made by measuring the optical density at 600 nm (OD_600_) using an UV-Vis spectrophotometer (Thermo Fisher Scientific, Stockholm, Sweden).

For the further experiments, the microorganisms were harvested by taking 2 mL aliquots of cultures and centrifuge at 13,200×*g* for 5 min. After removal of the supernatant, the pellets were washed two times with 2 mL 0.4% NaCl to remove remaining cultivation broth. The pellets were then subjected to the sample preparation described below.

### Sample preparation

**Fig. 2:**
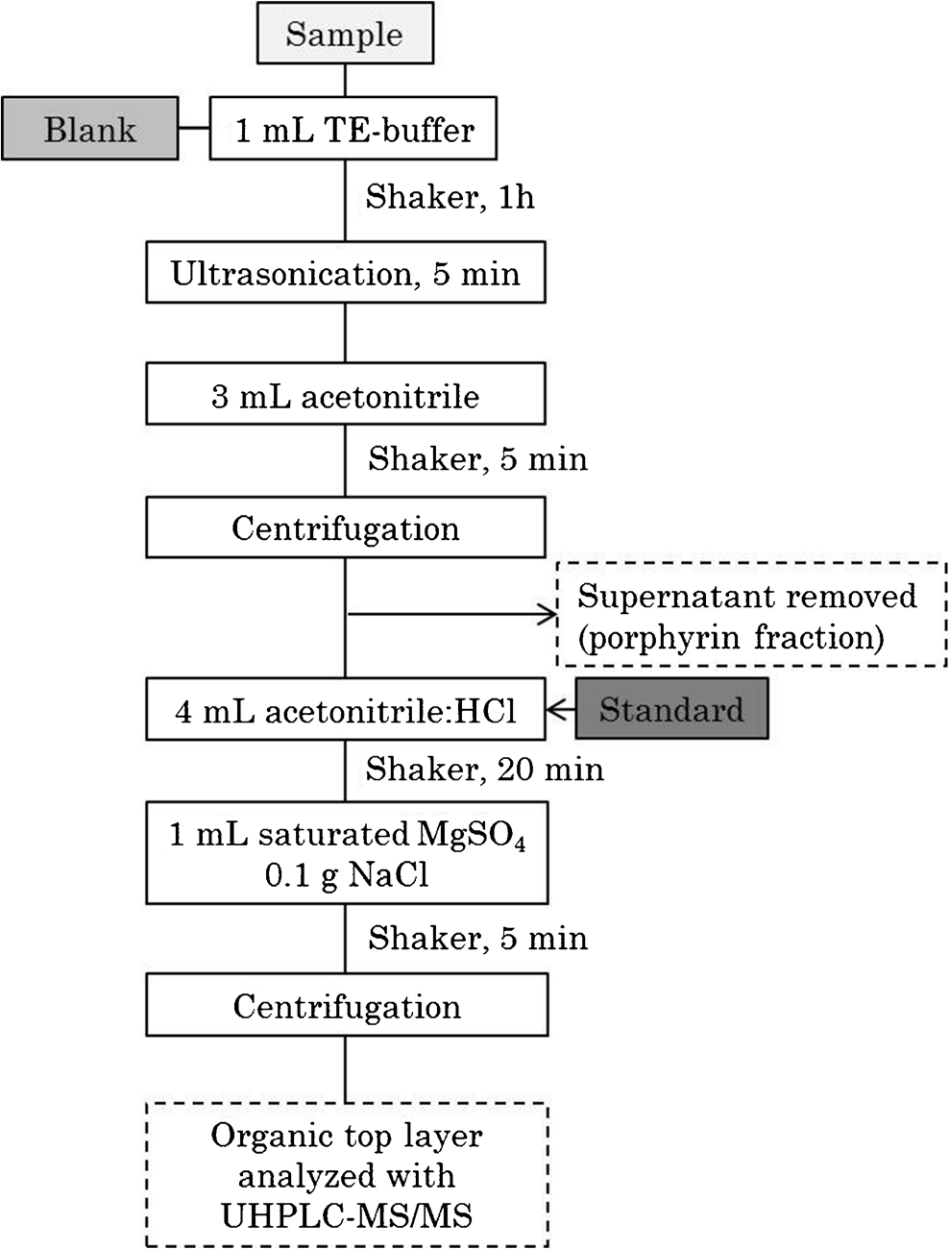
Scheme of the extraction and clean-up method. “Standard” points out were in the clean-up process the standard of heme for quantification purposes were added

Approximately 100 mg (wet weight) of *S. cerevisiae* and 3 mg (dry weight) of *E. coli* were put in 15-mL Falcon™ tubes together with 1 mL of Tris-EDTA buffer (pH 7.2) and stirred for 1 h at room temperature using a shaker (IKA VXR Vibrax, Staufen, Germany) at 1600 RPM. Samples were put on ice and treated with ultrasonication (Sonics Vibracell, Newtown, CT, USA) for 5 min with 1 s pulse. Three milliliters of acetonitrile was added to the sample which then was vortexed for 5 min and subsequently subjected to centrifugation at 2500×*g* for 5 min, making a pellet of precipitated proteins. The acetonitrile containing unpolar interfering compounds, as well as porphyrins, was removed and can be analyzed for porphyrin content. Then, 4 mL of acetonitrile:1.7 M HCl (8:2, *v*/*v*) was added to the pellet and put in a shaker for 20 min, extracting heme from the proteins into the acetonitrile. To create a two phase liquid-liquid system, 1 mL of saturated MgSO_4(aq)_ and 0.1 g of NaCl_(s)_ were added. The solution was vortexed for 5 min and centrifuged at 2500×*g* for 5 min. The top organic layer was put in a vial and, if necessary, diluted with pure acetonitrile prior to analysis. A blank and a standard used for quantification purposes were subjected to the same extraction and clean-up steps to rule out any contamination during clean-up and correct for losses. Figure 2 shows a scheme of the workflow.

### LC-MS/MS analysis

The analyses of heme, hemin, and porphyrins were performed on a Xevo TQ-S tandem mass spectrometer (MS/MS) coupled to a high performance liquid chromatograph Acquity I-Class system (Waters). An ACE 3 C18 column (2.1 × 50 mm, Advanced Chromatography Technologies Ltd., Aberdeen, Scotland), placed in a column oven (50 °C), was used for the separation with a gradient elution using water and acetonitrile with 0.1% formic acid (FA) as mobile phases. Signals from all analytes were acquired in positive electrospray mode (ESI+) and multiple reaction monitoring (MRM) with three compound-specific transitions for each analyte, all transitions used as quantifier and qualifier ions. The mass spectrometer was tuned by direct infusion of a standard solution with a concentration of 2 μM of the analytes. The dwell time for each transition was automatically set by the software to be 0.012 s in order to get approximate 12 data points over a peak with a width of 4 s.

Liquid chromatography and mass spectrometry parameters are presented in Table 2.

**Table 2:** Conditions for the LC-MS/MS analyses

HPLC		ESI-MS/MS conditions			
Chromatographic conditions		Electrospray conditions			
Column dimensions	2.1 × 50 mm	Ionization mode	ESI (+)		
Stationary phase	C-18	Capillary voltage	3500 V		
Particle size	3 μm	Source temperature	150 °C		
Column temperature	50 °C	Desolvation temperature	650 °C		
Mobile phase A	ACN/H_2_0	Desolvation gas flow	650 L/h		
	5:95 + 0.1% FA				
Mobile phase B	ACN/H_2_0				
	95:5 + 0.1% FA				
Flow rate	1 mL/min				
Injection volume	5 μL				
Autosampler temperature	5 °C				
Gradient		MS/MS conditions			
Time [min]	B%	Dwell time	0.012 s		
Initial	20	MS/MS	MRM		
5.00	100				
5.50	100	Compound	Transition	CV	CE
5.51	20	Uroporphyrin	831 > 727	10	112
6.00	20		831 > 623	10	112
			831 > 655	10	112
		7-Carboxylporphyrin	787 > 623	8	118
			787 > 682	8	120
			787 > 670	8	120
		6-Carboxylporphyrin	743 > 639	18	110
			743 > 507	18	110
			743 > 521	18	110
		5-Carboxylporphyrin	699 > 463	4	120
			699 > 495	4	108
			699 > 639	4	90
		Coproporphyrin	655 > 522	10	120
			655 > 536	10	120
			655 > 595	10	102
		Hemin	616 > 482	64	120
			616 > 497	64	116
			616 > 557	64	82
		Mesoporphyrin	568 > 478	2	102
			568 > 508	2	74
			568 > 449	2	74
		Protoporphyrin	563 > 444	20	110
			563 > 489	20	96
			563 > 504	20	78

*CV* cone voltage, *CE* collision energy

### Linearity and limit of detection

An 11-point calibration curve of hemin was made by injections of a standard solution in the range of 5–250 pmol to investigate the linear relationship between analyte concentration and instrumental response. Each level was injected in triplicate and analyzed in MRM mode. Limit of detection (LOD) was determined by injections of 0.75 pmol hemin (*n* = 7). The signal (*S*) was defined as the mean area of these replicate injections and the noise (*N*) defined as the standard deviation. Limit of detection were defined as three times the signal to noise ratio (*S*/*N*).

### Optimization of heme extraction

A full factorial central composite face design with three center points was used to optimize the extraction conditions of heme from *S. cerevisiae* with acetonitrile as the organic solvent*.* The evaluated factors were time of extraction (1, 20, and 40 min), percentage of acetonitrile (33, 66, and 99%, *v*/*v*), and molar concentration of HCl (0.1, 1, and 2 mol/dm^3^) using 17 experiments. A total weight of 0.8 g *S. cerevisiae* was put in 3 mL of Tris/EDTA buffer for digestion during 1 h and further lysed by ultrasonication for 5 min (1 s pulse). Aliquots of 100 μL were transferred to 0.5 mL micro vials and 400 μL of different acetonitrile/HCl mixtures was added and was immediately vortexed for 1, 20, or 40 min. Immediately after vortexing, the micro vials were centrifuged at 13,200×*g* and the supernatant transferred to vials and analyzed for heme content. The experimental data were processed using MODDE software (ver. 10.0.0; MKS Umetrics, Umeå, Sweden).

### Protein precipitation efficiency

Five different solvent mixtures were selected for evaluation of their ability to precipitate proteins from *S. cerevisiae*. Acetone, acetonitrile, and methanol solutions were mixtures of 80% organic solvent together with 20% 1.7 M HCl (*v*/*v*). Pure acetonitrile as well as 1.7 M HCl were also evaluated.

*S. cerevisiae* was lysed as described above, and 100 μL yeast extract was added to 200 μL of precipitation solvent in 1.5 mL micro vials, vortexed for 20 s, and left to stand for 10 min. Solutions were centrifuged at 13,200×*g* for 5 min. Measurement of the protein concentration was done with a spectrophotometer (Waters) using the Bradford micro protein assay at a wavelength of 595 nm. Absorbances before and after protein precipitation were used to calculate the protein precipitation efficiency by the equation;

$$ \frac{\mathrm{Absorbance}\  \mathrm{before}\  \mathrm{precipitation}-\mathrm{Absorbance}\  \mathrm{after}\  \mathrm{precipitation}}{\mathrm{Absorbance}\  \mathrm{before}\  \mathrm{precipitation}}\times 100\% $$

Bovine serum albumin standards were used to check the linear response of the spectrophotometer. The experiments were performed in triplicate with three absorbance measurements of each sample.

### Stability of heme in different solvents

Storage stability of hemin in room temperature was investigated using six solvents and solvent mixtures: 100% acetonitrile, 1 M NaOH, 6 M formic acid, deionized water, acetonitrile:1.7 M HCl (8:2, *v*/*v*), and the organic top layer from the liquid-liquid extraction when using acetonitrile as described above. Hemin standards dissolved in the six solvents were put in the autosampler tray and analyzed daily in duplicate during 7 days. For each analytical run, a volumetric standard was used to account for day to day variations in instrumental response.

## Results and discussion

### Optimization of heme extraction

Acetone extraction has been used to extract heme from sample matrices such as plants [20, 23] and algae [24] and is generally adopted as the standard procedure for heme extraction. The extractions are normally carried out in 80% acetone containing 20% of 0.6–2.1 M HCl [20]. However, acetonitrile has several advantages compared to acetone as a solvent. It has lower elution strength in reversed phase LC columns, making it more suitable to inject without extensive band broadening of the peaks on reversed phase columns. It also has a lower UV cutoff wavelength, making it more suitable for the detection with UV-vis, and it has been shown to possess better protein precipitation properties when the aim has been to remove proteins from the sample matrix [25]. For these reasons, acetonitrile was selected as a candidate solvent for optimization of heme extraction.

**Fig. 3:**
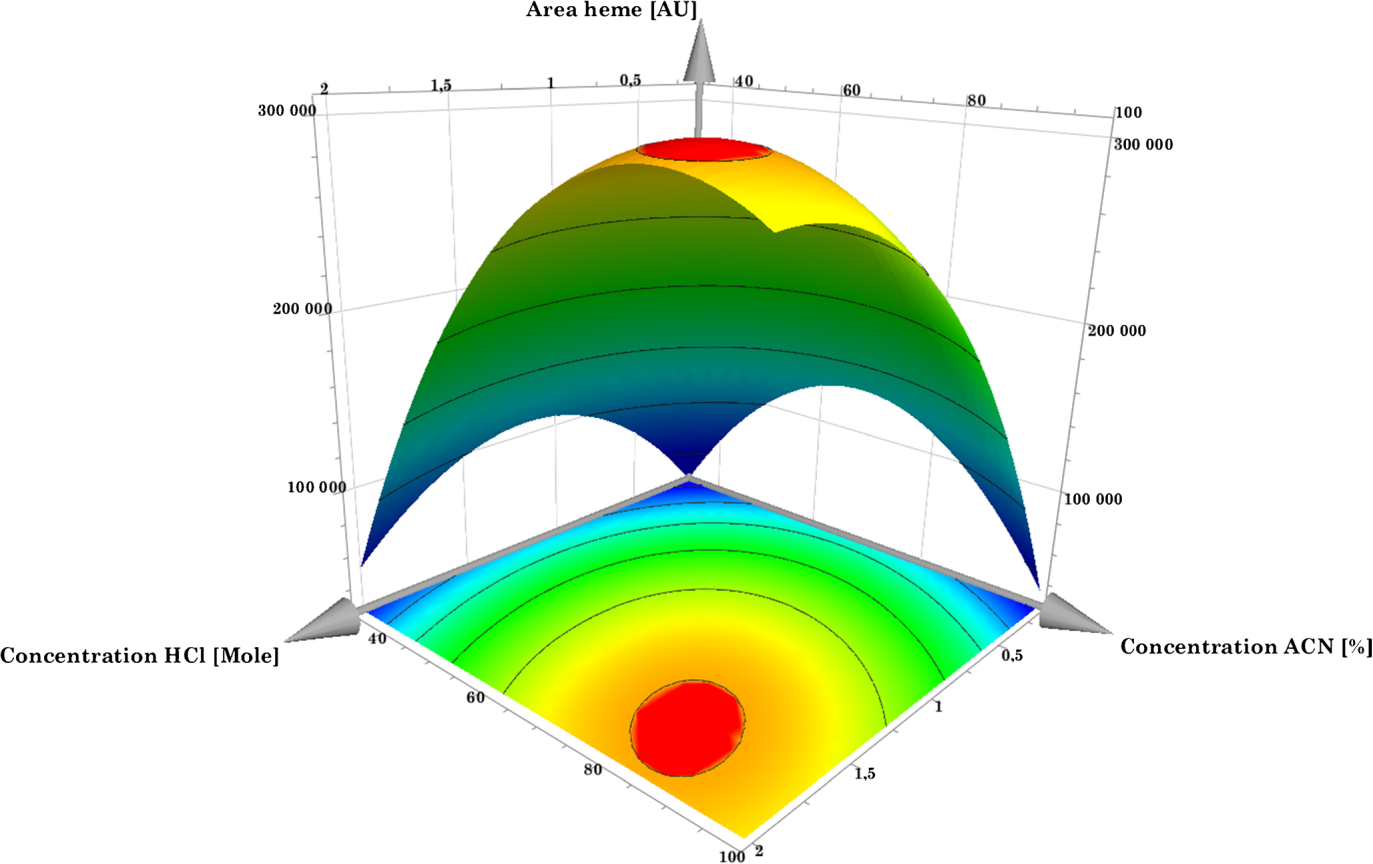
Response surface plot of heme extraction at 20 min, with concentration of acetonitrile versus concentration of HCl. Red color marks the region of highest response

The experiments were evaluated using the MODDE software, and the coefficient plot showed that only two factors were significantly affecting the extraction efficiency of heme (*p* ≤ 0.05): the molar concentration of HCl and the percentage of acetonitrile. Surprisingly, the extraction time was found not to influence the extraction efficiency of heme within the experimental domain (1–40 min). There was a weak trend that more heme was extracted when longer extraction times were used, but the differences were small and not significant. In Fig. 3, the concentrations of both acetonitrile and HCl are plotted in a response surface plot when samples were extracted for 20 min. Both HCl and acetonitrile concentrations have a positive effect on the heme extraction with an optimum in the selected domain. Trying to extract heme into pure acetonitrile, not containing any HCl, resulted in no detectable amounts of heme. Using only 1.7 M HCl resulted in poor yield of heme, < 0.6%. A possible explanation to this is that when the extraction is performed in 80% acetonitrile, there will be no protonation of the heme binding amino acids and heme will still be bonded to the proteins. When HCl, or another strong acid, is used together with the organic solvent, heme is released from these proteins. On the other hand, when only HCl is used, heme is too hydrophobic to be extracted from the precipitated proteins and into the polar solvent.

The optimum in this model was determined to be 82% acetonitrile and 18% 1.7 M HCl when using 20 min extraction time. These results are in agreement with previous studies where other authors have used acetone and HCl for the extraction [20, 23, 26, 27]. The precipitated yeast pellet was subjected to repeated extractions with the optimized method. In the second extraction, 3.3% of the amount found in the first extraction was detected. In the third extraction, less than 1% was found, i.e., after two extractions of the sample the yield was considered to be > 99%.

**Fig. 4:**
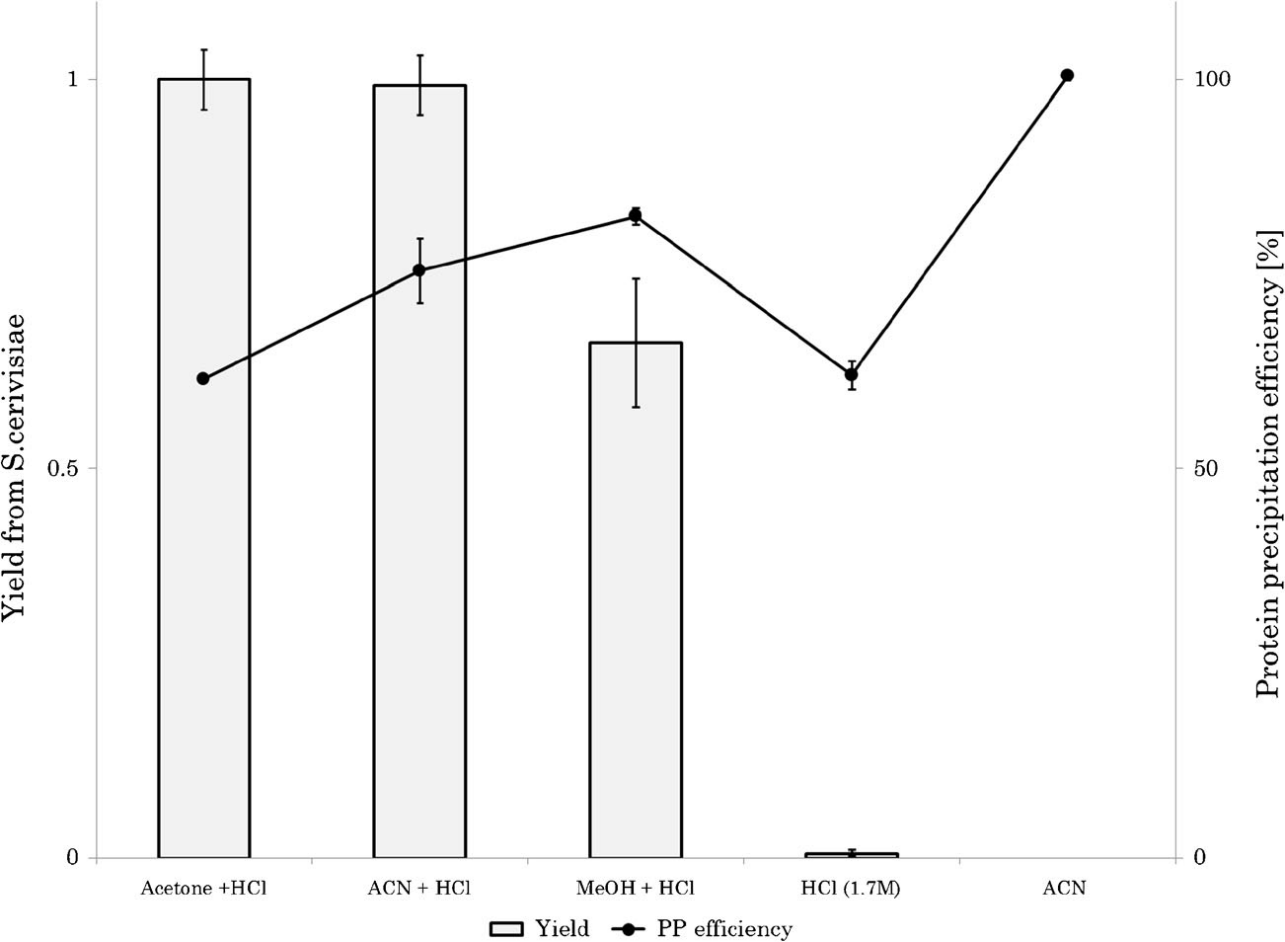
Relative yield (bars) of heme from *S. cerevisiae* using different extraction solvents and their protein precipitation efficiency (lines). Error bars represent the standard deviation for triplicate

When using methanol with 20% 1.7 M HCl, as well as pure 1.7 M HCl for the extraction, the efficiencies were much lower compared to using acetonitrile/HCl. Methanol/HCl had a relative extraction efficiency of 66% and 1.7 M HCl only had 0.6%. Acetone:1.7 M HCl, on the other hand, demonstrated similar extraction capabilities as acetonitrile:1.7 M HCl. Methanol is a more polar solvent compared to acetone and acetonitrile and 1.7 M of HCl being the most polar of the investigated solvents. This explains the lower extraction yields when using these solvents. When using acetonitrile without any HCl, the heme level in the extract was below LOD. The yield using the different solvents, together with the protein precipitation efficiencies, is shown in Fig. 4.

### Protein precipitation

Analysis of samples containing high concentrations of proteins often requires clean-up to reduce matrix effects in the LC-MS/MS analysis. In Fig. 4, the protein precipitation efficiencies of the solvent mixtures are shown. The most efficient precipitant was 100% acetonitrile which precipitated 100.5% (± 0.6) of the proteins present in the *S. cerevisiae* extracts. This is in agreement with other studies where acetonitrile has been reported to be the most efficient precipitant to proteins in blood plasma [25, 28]. Methanol:1.7 M HCl (8:2, *v*/*v*) precipitated 82.4% (± 1.0) of the total protein content, while acetonitrile:1.7 M HCl (8:2, *v*/*v*) had a precipitation efficiency of 75.4% (± 4.1). Acetone:1.7 M HCl (8:2, *v*/*v*) and 1.7 M HCl had the lowest protein precipitation efficiency with 61.5% (± 0.2) and 62.0% (± 1.8), respectively. All determinations of protein precipitation efficiencies are average values from three replicates.

### Clean-up and stability of heme

One of the most crucial problems to overcome in heme analysis utilizing LC-MS/MS instrumentation is the ability of heme to form aggregates and precipitate in aqueous solutions [29, 30]. When a standard of hemin was analyzed after having been dissolved in MQ water and left for 24 h at room temperature, the hemin concentration had decreased with 28%. Increasing as well as decreasing the pH with NaOH and formic acid made hemin aggregate quicker. After 24 h in 1 M of NaOH and in 6 M formic acid, the hemin content had decreased to 52 and 45%, respectively. This makes the analysis of heme from microorganisms problematic since the optimized extraction solvent requires the addition of an acid in order to extract heme from the heme proteins. When hemin was stored in 100% of acetonitrile, it was more stable and had only decreased 6% after 1 week of storage in room temperature. However, when hemin was put in the solvent composition used for the optimized extraction procedure (acetonitrile:1.7 M HCl (8:2, *v*/*v*)), it aggregates and the concentration decreased to 85% within 24 h, and after 1 week, only 49% of the hemin content remained un-aggregated.

The poor yield of heme into pure acetonitrile can be used in the clean-up process of microbiological samples. Adding 100% acetonitrile to the lysate will remove hydrophobic interfering substances after centrifugation and removal of the supernatant (porphyrin fraction). Heme will still be bond to the sedimented proteins and can subsequently be extracted with the optimized extraction solvent.

**Fig. 5:**
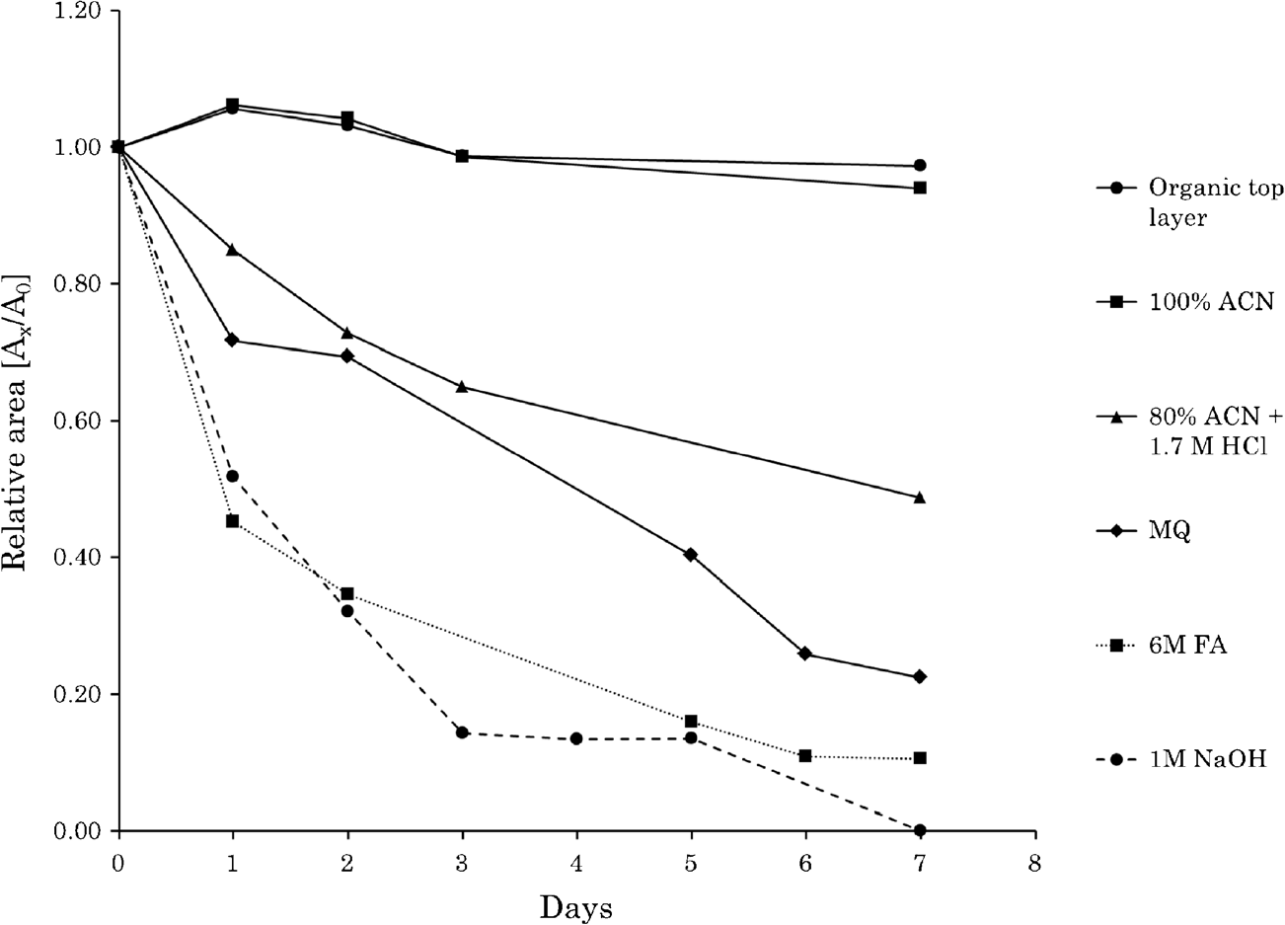
Hemin stability during 7 days when dissolved in six different solvents. A_x_ is the area ratio to a volumetric standard at the investigated day and A_0_ is the area ratio to a volumetric standard at day 0. Organic top layer is the solvent used in the liquid-liquid extraction

To stabilize heme in the extraction solvent, 1 mL of saturated MgSO_4(aq)_ and 0.1 g of NaCl_(s)_ were added. A two-phase system with acetonitrile in the top layer and the aqueous solution in the bottom layer was formed. Polar interfering substances distributed into the aqueous layer and were removed from the sample, while heme partitioned into the organic solvent. No heme could be detected in the lower aqueous layer. A stability test of hemin dissolved in this organic top layer solution showed no decrease of hemin after 1 week of storage in room temperature. The stability of hemin in all the tested solvents is shown in Fig. 5.

Methods for determination of heme in various biological matrices have been presented in the literature. However, the aggregation and precipitation of heme has not been considered and/or the analytical methods lack sufficient validation [31–35]. This raise questions for the accuracy in reported values of heme concentrations. The method presented here has solved the problem with heme aggregation and precipitation demonstrated by stability during at least 1 week of storage in room temperature. Further evaluation is needed if samples are stored for a longer time and/or at higher as well as lower temperatures.

### Analysis of heme with HPLC-MS/MS

#### Chromatographic separation

**Fig. 6:**
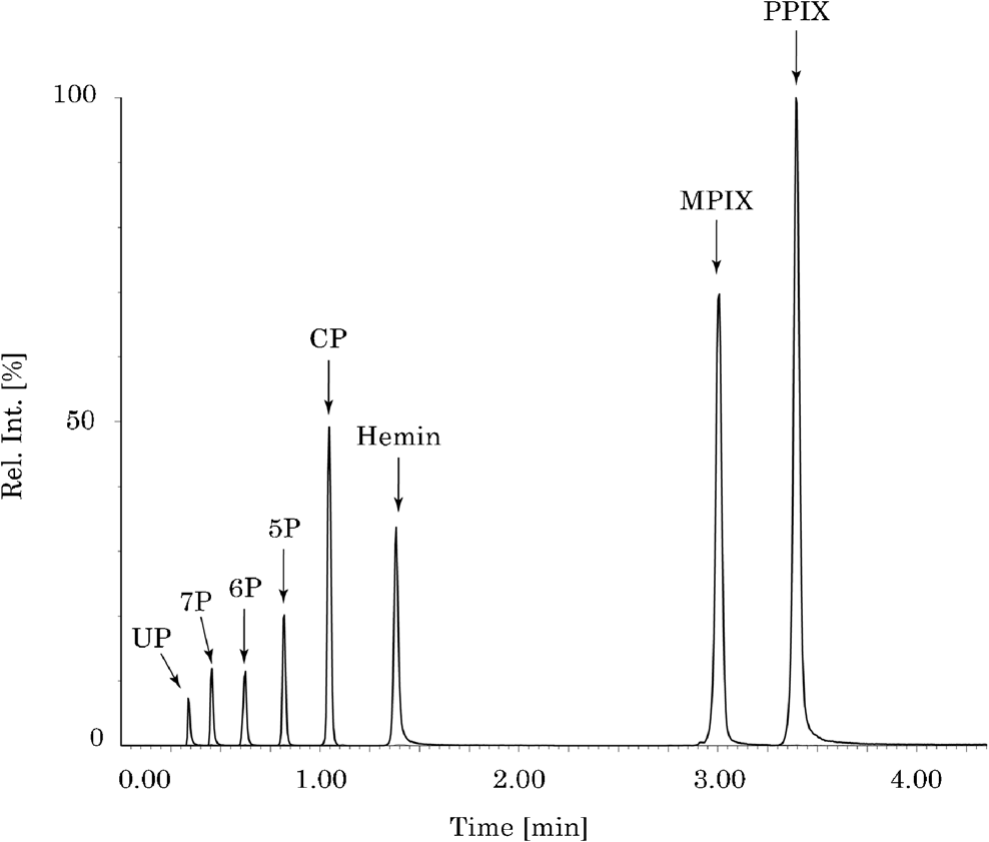
Total reconstructed ion chromatogram from the HPLC-MS/MS analysis of a standard mixture of porphyrins and hemin. UP, uroporphyrin; 7P, 7-carboxylporphyrin; 6P, 6-carboxylporphyrin; 5P, 5-carboxylporphyrin; CP, coproporphyrin; Hemin; MPIX, mesoporphyrin IX; PPIX, protoporphyrin IX. Flow rate = 1 mL/min. Injection volume = 5 μL. Detection: MRM using three compound-specific transitions for each analyte

In Fig. 6, a chromatogram is shown for the HPLC-MS/MS analysis of hemin and heme precursors (porphyrins). All compounds are baseline separated within 4 min. Total runtime for one sample, including pre- and post-runs to clean and condition the column, is 6 min. In other methods where heme has been separated with HPLC, the chromatographic run normally takes between 15 and 45 min [22, 33, 36, 37].

#### Linearity and detection limit

The calibration curve showed nonlinear characteristics with a significant curvature in the concentration range 5–250 pmol. This pattern of hemin linearity was demonstrated on two different mass spectrometers with electrospray ionization and is probably explained by signal saturation in the electrospray at high concentrations [38]. To be able to model the data in in this concentration range, it is recommended to use a polynomial curve fitting of second degree (*R* ^2^ > 0.99). A linear calibration curve (*R* ^2^ > 0.98) was obtained when limiting the concentration range to 5–100 pmol (Fig. S1 in Electronic Supplementary Material (ESM)).

Limit of detection was determined at 0.2 pmol of injected hemin. The LOD is the lowest concentration of an analyte that the analytical process can reliably differentiate from background levels [39]. A low LOD is of importance when applying this method to other microorganisms that are not as easy to culture as *S. cerevisiae*, thus yielding smaller sample sizes. We have not been able to find any reported LOD values from previous studies, but we consider 0.2 pmol to be low for this kind of matrices.

#### Matrix effect

Approximately 150 mg (wet weight) of *S. cerevisiae* was put through the sample preparation steps with the only difference that the acetonitrile used in the liquid-liquid extraction did not contain any HCl, thus not extracting any heme from the yeast cells. In this way, an analyte-free matrix was created close to a real sample matrix. The analyte-free matrix was spiked with hemin to a concentration of 15 μM. The response obtained from the HPLC-MS/MS analysis was compared to a reference standard dissolved in pure extraction solvent. The results showed that the matrix effect was low, with a value of 106 ± 3% which was not significantly different from 100% (*p* ≤ 0.05). The low matrix effect demonstrates that the clean-up procedure is efficient in removing interfering compounds, and the reproducibility and accuracy could be considered as high.

#### Precision and recovery

The intraday precision was determined at three different concentration levels, 0.015, 0.15, and 15 μM, in an analyte-free matrix produced as described above. Each concentration level was injected in triplicate and the average intraday variation was low and found to be 6 ± 5%.

Three different concentration levels, 0.15, 1.50, and 15.00 μM, were put through the clean-up steps described above to determine the recovery. The signal responses were compared to a standard dissolved in the same solvent composition. The average recovery for the three different concentration levels was found to be 89 ± 9%.

### Application of the method

#### Determination of heme in *S. cerevisiae*

To demonstrate the applicability of the developed method, samples of approximately 100 mg (wet weight) of *S. cerevisiae* were put through the extraction and clean-up steps described in the “Experimental” section. Heme was identified and quantified in all the samples with an average concentration of 51 ± 5 nmol/g. A chromatogram of heme analysis in *S. cerevisiae* can be seen in ESM Fig. S2. When the porphyrin fraction in the liquid-liquid extraction was analyzed, two isomers of coproporphyrin (I and III) and protoporphyrin IX were detected in all samples.

#### Effect of additives and time of cultivation on the heme concentration in *E. coli*

**Fig. 7:**
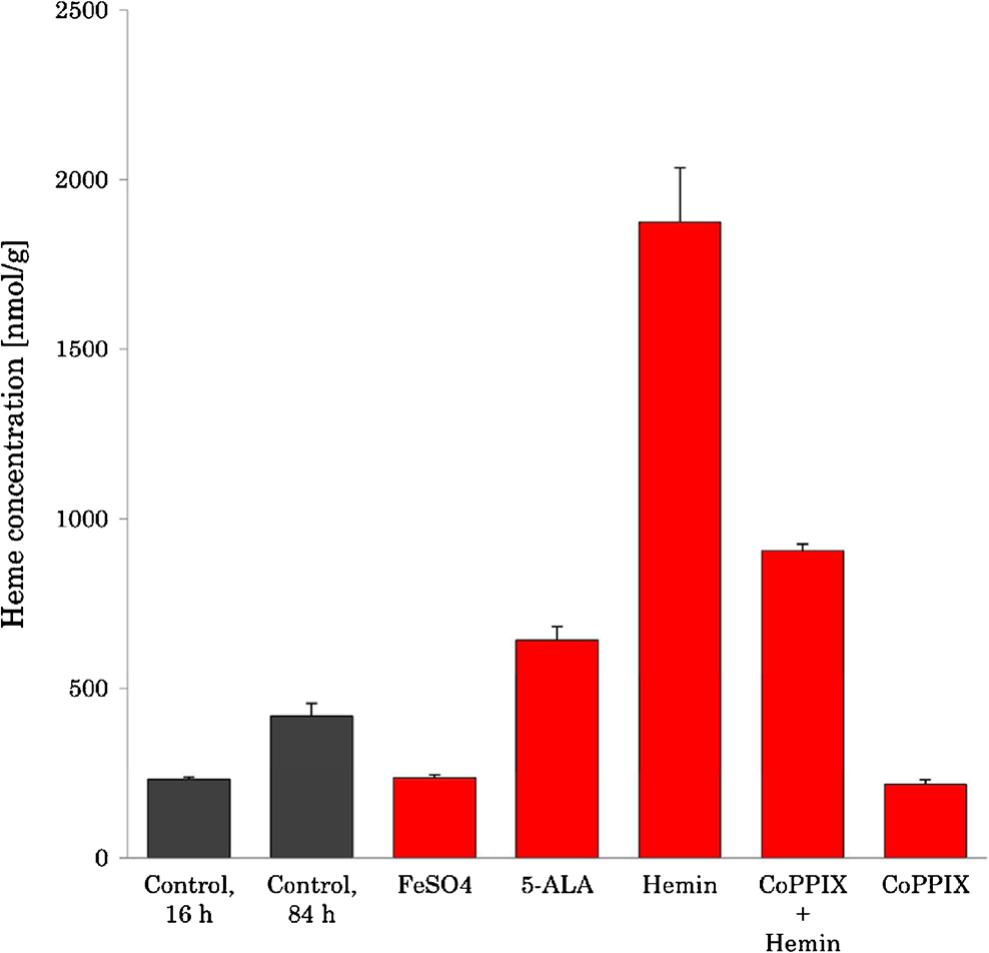
Heme concentrations in *E. coli* with different culturing conditions

When *E. coli* was analyzed with different additives and culturing length, the heme concentrations were shown to be affected (Fig. 7). Addition of Fe^2+^ ions added to the LB broth had however no significant increase in heme concentrations (235 ± 10 nmol/g) compared to the control (231 ± 7 nmol/g). The length of culturing time has previously been shown to affect the concentration of heme and porphyrins in studies of other microorganisms [40, 41]. When culturing *E. coli* for 84 h, the heme concentration increased to 418 ± 37 nmol/g and compared to the control cultured for 16 h. This corresponds to an increase in the heme concentration with a factor of 1.8.

**Fig. 8:**
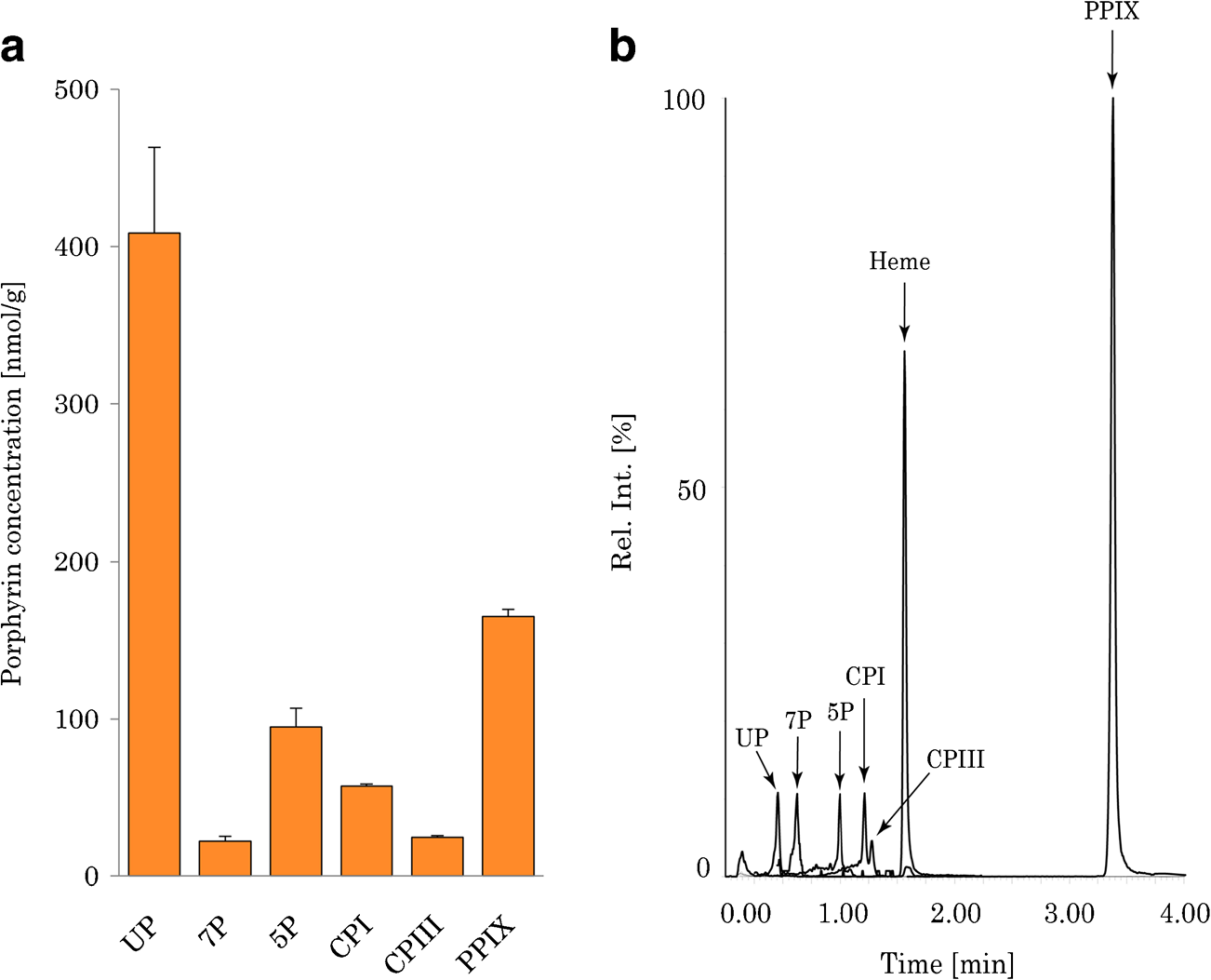
Porphyrin concentrations (**A**) and HPLC-MS/MS chromatogram (**B**) of a sample after addition of 5-ALA to *E. coli* growth medium. Due to the high response of protoporphyrin IX, the chromatogram has been enlarged in the range 0.00–1.50 min to make the other peaks visible. UP, uroporphyrin; 7P, 7-carboxylporphyrin; 5P, 5-carboxylporphyrin; CPI, coproporphyrin I; CPIII, coproporphyrin III; PPIX, protoporphyrin IX

The de novo synthesis of heme is tightly regulated by a negative feedback control. High heme concentrations inside the cell will inhibit the 5-aminolaevulinic acid synthase activity which is the rate-limiting step in heme biosynthesis. Adding exogenous 5-ALA to the growth medium will bypass the negative feedback control, and if the bacteria have all the necessary enzymes for heme biosynthesis, a higher concentration of heme is expected. When *E. coli* was grown in a medium with 5.0 mM of 5-ALA, the heme concentration was 642 ± 41 nmol/g, which is a 53% increase compared to the 84-h control (*p* ≤ 0.05). This results show that *E. coli* has the ability to synthesize heme from 5-ALA, and this is further emphasized when the bacterial extracts were analyzed for porphyrins. In all the other bacterial experiments, only protoporphyrin IX and coproporphyrin could be detected at low concentrations. When 5-ALA was added to the growth medium, uroporphyrin, 7-carboxylporphyrin, 5-carboxylporphyrin, coproporphyrins I and III, as well as protoporphyrin IX were detected in high concentrations (Fig. 8).

Hemin was added to the growth medium to investigate if *E. coli* has the ability to acquire heme from its surroundings. When grown in a heme-enriched medium, the heme concentration in *E. coli* increased substantially compared to the control. A 4.5-fold increase in heme concentration was observed, reaching a concentration of 1874 ± 161 nmol/g. The bacterial pellets were washed twice with NaCl solution prior the clean-up process in order to remove any heme-containing broth and the final wash solution was analyzed for heme. No heme could be detected in the last washing solution. High concentrations of heme in these samples show that *E. coli* has a high affinity for exogenous heme.

#### Cobalt protoporphyrin IX inhibition of heme acquisition

Cobalt protoporphyrin IX has been shown to exhibit antimicrobial activity against *Porphyromonas gingivalis*, reducing both planktonic and biofilm growth [9]. The Co-PPIX molecule is identical to heme with the exception that iron(II) in the center of the heme molecule has been replaced with cobalt(III). Cobalt protoporphyrin IX could chemically mimic heme in heme-acquiring microorganisms, implying that addition of Co-PPIX could have the potential to disturb the heme metabolism in bacteria similar to *E. coli.* Cobalt protoporphyrin IX and hemin were added in equimolar concentrations (10 μM each) to the cultivation broth. When the bacterial extracts were analyzed, a reduction with 52% (905 ± 20 nmol/g) was observed, which was equivalent to the molar ratio of hemin/Co-PPIX in the cultivation broth. A reduction with 48% in heme concentrations was also observed when *E. coli* was cultured only with Co-PPIX as an additive (217 ± 14 nmol/g). This demonstrates that the *E. coli* heme-acquiring systems have the same affinity for Co-PPIX as for heme which potentially makes Co-PPIX a good candidate to chemically mimic heme. To investigate if Co-PPIX is taken up by the bacteria, all bacterial extracts were additionally analyzed in full scan mode with a mass range between 100 and 1500 Da. When the [M+H]^+^ ion of Co-PPIX (*m*/*z* 618.4) was extracted and verified by the chromatographic retention time with a Co-PPIX standard, it was shown that Co-PPIX was only present in the extracts of *E. coli* in which the medium had been spiked with Co-PPIX (ESM Fig. S3).

However, even though these results demonstrate that the heme-acquiring system of *E. coli* confuses Co-PPIX with heme and has an uptake of Co-PPIX, no antimicrobial effect was observed. Starting cultures of *E. coli* were allowed to grow in broth spiked with Co-PPIX, and after 24 h, no statistical differences in bacterial number compared to a control could be seen.

There could however be an antimicrobial effect if higher concentrations of Co-PPIX are used, and potential antimicrobial effect of Co-PPIX to *E. coli* should be studied in more detail regarding concentrations of Co-PPIX and different cultivation media to gain more knowledge about *E. coli* susceptibility against Co-PPIX.

## Conclusions

By combining a selective, but still simple, liquid-liquid extraction with a selective HPLC-MS/MS analysis and applying experimental design to find optimal extraction conditions, a method for determination of heme as well as heme precursors, i.e., porphyrins, in microorganisms has been developed. When this method was applied on *S. cerevisiae* and *E. coli*, it was shown that the heme concentrations were affected by using different additives in the cultivation media. Furthermore, it was shown that cobalt protoporphyrin IX was able to mimic heme and was taken up by *E. coli*, leading to a reduction in intracellular concentrations of heme. This result indicates that heme-acquiring mechanisms in microorganisms could potentially be a good drug target to treat bacterial infections.

However, no antimicrobial effect of Co-PPIX on *E. coli* could be shown, but this will be a subject to further investigations.

## Electronic supplementary material

ESM 1 (PDF 640 kb)

## Abbreviations

5-ALA: 5-Aminolevulinic acid hydrochloride
Co-PPIX: Cobalt protoporphyrin IX
LB: Lysogeny broth
OD_600_: Optical density at 600 nm
