# Supplementary material for: Determination of heme in microorganisms using HPLC-MS/MS and cobalt(III) protoporphyrin IX inhibition of heme acquisition in Escherichia coli
Source: Anal Bioanal Chem. 2017 Oct 17;409(30):6999–7010. doi: 10.1007/s00216-017-0610-5 (PMC5717118; doi:10.1007/s00216-017-0610-5)
Supplement: Supplementary file 1 — (PDF 640 kb) [file 216_2017_610_MOESM1_ESM.pdf]

Analytical and Bioanalytical Chemistry

Electronic Supplementary Material

Determination of heme in microorganisms using HPLC-MS/MS and cobalt(III) protoporphyrin IX inhibition of heme acquisition in *Escherichia coli*

Jonas Fyrestam, Conny Östman

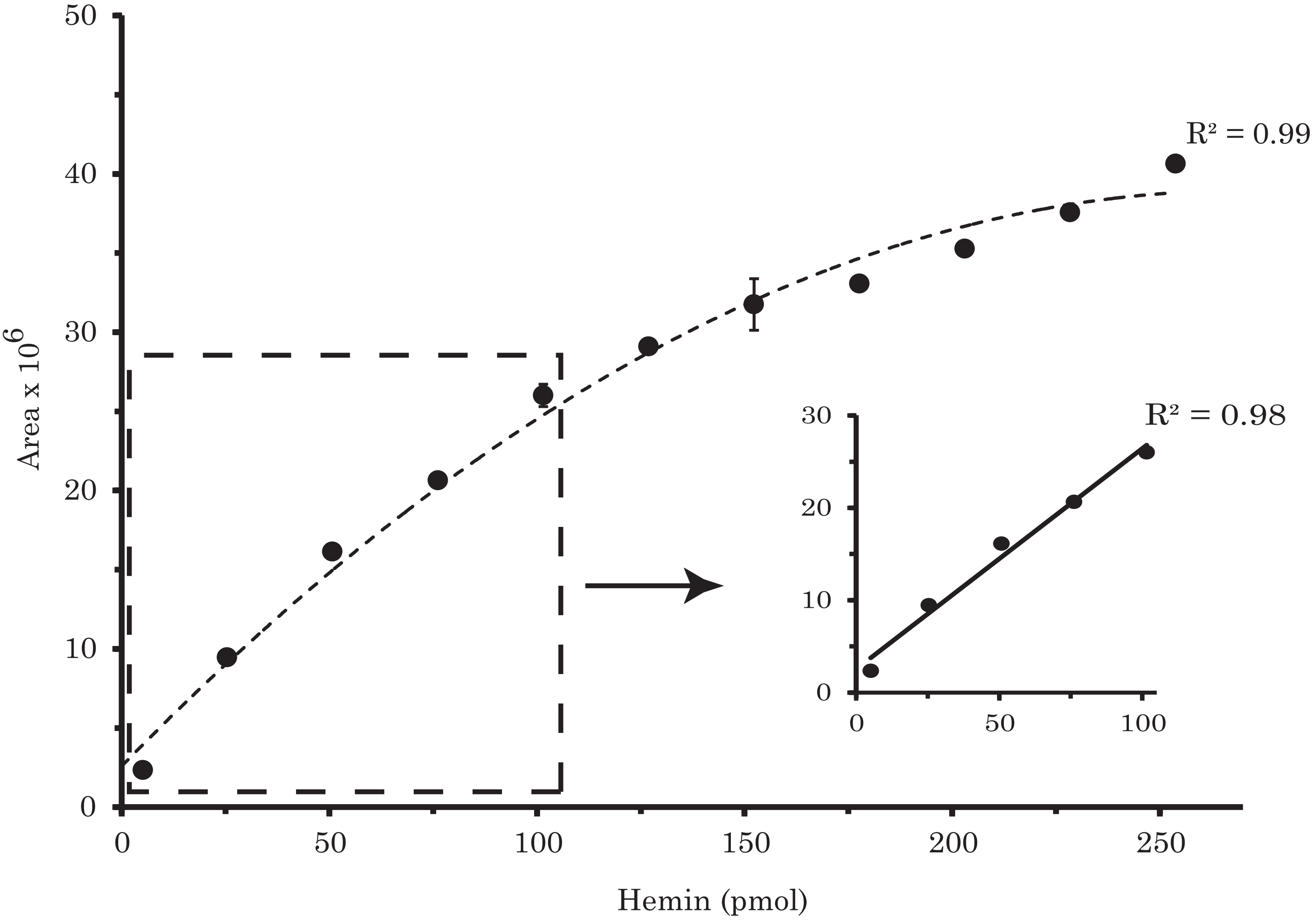

Fig. S1 Calibration curve for hemin at 11 different levels (5-250 pmol). The dashed regression line is a second degree polynomial fitted to all data points (n=11). Solid linear regression line is based on the five lowest levels, 5-100 pmol. Each data point is an average of triplicate injections except point 5 and 7 were n=2

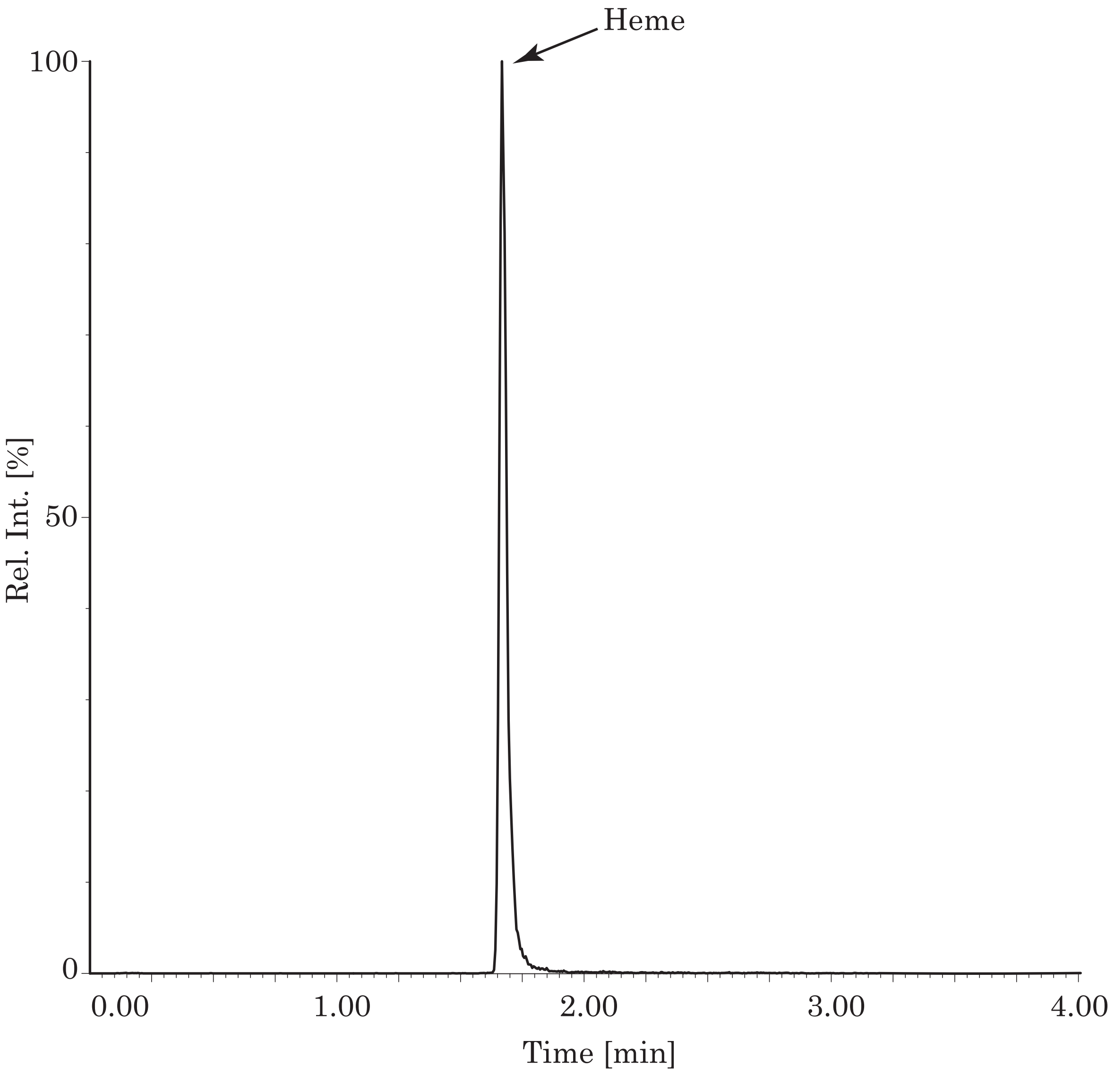

Fig. S2 Total ion chromatogram of *S. cerevisiae* extract

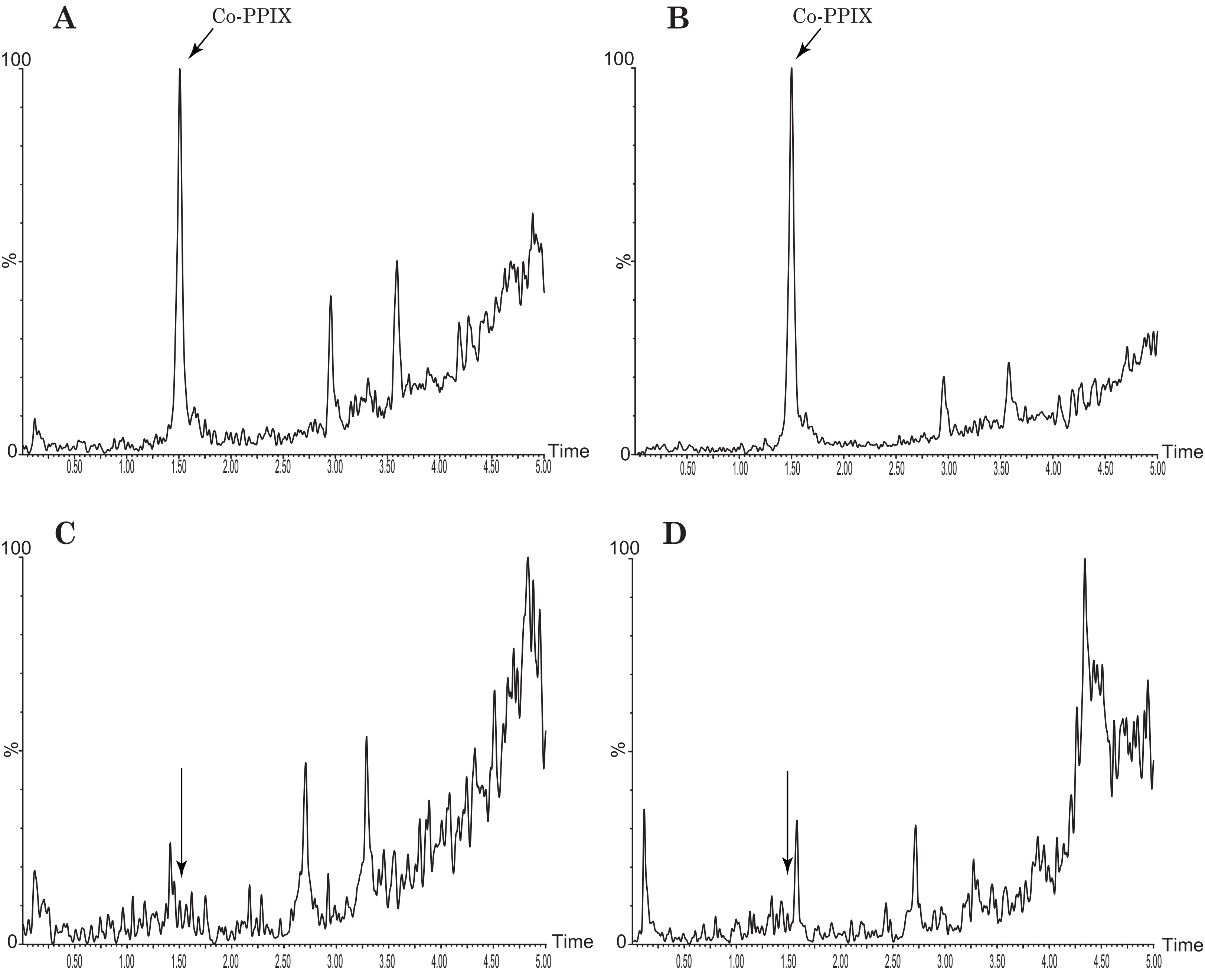

Fig. S3 Extracted ion chromatogram comparisons of Co-PPIX (m/z 618.4 Da) from four individual *E. coli* extracts where different additives have been added to the growth medium; (A) cobalt protoporphyrin IX, (B) hemin and cobalt protoporphyrin IX, (C) 5-aminolaevulinic acid and (D) hemin. The arrows in chromatograms (C) and (D) show the retention time for Co-PPIX
